# Medica Sacra; or, Short Expositions of the Most Important Diseases Mentioned in the Sacred Writings

**Published:** 1834-07-01

**Authors:** 


*Medica Sacra; or, Short Expositions of the most important Diseases mentioned in the Sacred Writings.* By THOMAS SHAPTER, M.D., Physician to the Exeter Dispensary and Lying-in Charity, &c.—London, 1834. Small 8vo. pp. 191.

DR. SHAPTER anticipates that

"some may say, that whilst there exists a work under the same title as this small production of mine, with the important prefix of Dr. Mead's highly and justly esteemed name, another was superfluous: sufficient apology, however, on this score may be offered; for independently of my dissent from many of the views of Dr. Mead on the diseases under discussion, it must be borne in mind, that the Medica Sacra of this learned physician was published in Latin, and that its preface contains the following anathema against those sufficiently bold to translate it.

"'Βεβήλοις autem hæc non scripsi; sed iis tantum, qui aut sacris theologicis, aut medicis, initiati sint et eruditi. Eâque de causâ Latino potissimum sermone in lucem edere placuit; quem per multa jam sæcula docti homines ad ea inter se communicanda, quæ aut nova, aut præter vulgarem opinionem dicta viderentur, ubique fere adhibuerunt. Si quis igitur libri hujusce Anglicam versionem suscipiat, non tantum, me invito, id se facturum sciat; sed etiam contra jus illud æquabile, quo de re sua, prout libeat, statuere unicuique conceditur.'" (*Pref.*, p. vi.)

In this passage our author seems to suppose that there is no translation of Mead's Medica Sacra, whereas it is to be found in the English edition of his works, printed in 1762, in one lusty quarto. If Dr. Shapter has not seen this translation, his little work affords an example of one of the most curious literary coincidences on record; for, at p. 186, in quoting Mead's opinion of Hezekiah's case, he uses the same words *(verbatim et literatim)* that were used by the former English translator, (p. 602, ed. 1762.)

This work is not discreditable to Dr. Shapter, for it shows liberal principles and some research; yet, were it not inconsistent with the liberty of our medical republic, we could wish that all physicians were prohibited from wandering into these by-paths, until they had worked on the high-roads of medicine. When a man can say, as Mead does in his preface,

"my declining years having in a great measure released me from those medical fatigues in which, for the public good, (at least as I hope,) I have been employed about fifty years," &c., we would willingly allow that he should pass his learned leisure as he pleased, like a gladiator *rude donatus*. Unfortunately, however, the medical press groans under books which have little more tendency to improve the diagnosis or the treatment of disease, than the novels of Smollett, or the poetry of Akenside.

